# Interaction With Social Robots: Improving Gaze Toward Face but Not Necessarily Joint Attention in Children With Autism Spectrum Disorder

**DOI:** 10.3389/fpsyg.2019.01503

**Published:** 2019-07-05

**Authors:** Wei Cao, Wenxu Song, Xinge Li, Sixiao Zheng, Ge Zhang, Yanting Wu, Sailing He, Huilin Zhu, Jiajia Chen

**Affiliations:** ^1^Centre for Optical and Electromagnetic Research, South China Academy of Advanced Optoelectronics, South China Normal University, Guangzhou, China; ^2^School of Psychology, South China Normal University, Guangzhou, China; ^3^Academy for Engineering and Technology, Fudan University, Shanghai, China; ^4^Caihongqiao Children Rehabilitation and Service Center of Panyu District, Guangzhou, China; ^5^Child Development and Behavior Center, Third Affiliated Hospital of Sun Yat-sen University, Guangzhou, China; ^6^KTH Royal Institute of Technology, Stockholm, Sweden

**Keywords:** autism spectrum disorder, social robot, joint attention, longest common subsequence, eye tracking

## Abstract

It is widely recognized that robot-based interventions for autism spectrum disorders (ASD) hold promise, but the question remains as to whether social humanoid robots could facilitate joint attention performance in children with ASD. In this study, responsive joint attention was measured under two conditions in which different agents, a human and a robot, initiated joint attention via video. The participants were 15 children with ASD (mean age: 4.96 ± 1.10 years) and 15 typically developing (TD) children (mean age: 4.53 ± 0.90 years). In addition to analyses of fixation time and gaze transitions, a longest common subsequence approach (LCS) was employed to compare participants’ eye movements to a predefined logical reference sequence. The fixation of TD toward agent’s face was earlier and longer than children with ASD. Moreover, TD showed a greater number of gaze transitions between agent’s face and target, and higher LCS scores than children with ASD. Both groups showed more interests in the robot’s face, but the robot induced a lower proportion of fixation time on the target. Meanwhile participants showed similar gaze transitions and LCS results in both conditions, suggesting that they could follow the logic of the joint attention task induced by the robot as well as human. We have discussed the implications for the effects and applications of social humanoid robots in joint attention interventions.

## Introduction

Autism spectrum disorder (ASD) is a neurodevelopmental disorder. Its overall prevalence is approximately one in 68 in North America (Christensen et al., 2016) and is similar in the developed region of China (Sun et al., 2015). Children diagnosed with ASD show persistent deficits in non-verbal communicative behaviors during social interaction, especially in maintaining eye contact and in understanding and following an interlocutor’s gaze direction (American Psychiatric Association, 2013).

Joint attention is one of the bases of social interactions; it constitutes a milestone in the early development of social communication, and is closely associated with language acquisition. Because this skill plays a key role in the developmental deficits of children with ASD, it has always been targeted by ASD interventions (Charman, 2003). For example, the Joint Attention, Symbolic Play, Engagement and Regulation (JASPER) approach cooperated both applied behavior analysis and developmental procedures (hierarchy prompts and reinforcement) to increase the ability to coordinate attention and teach joint attention skills directly (Kasari et al., 2006). Recently, increasingly advanced technologies, such as humanoid social robots, have become involved in the diagnosis of and interventions for ASD (Diehl et al., 2012). Humanoid robots can provide similar social cues to humans in interaction and communication, such as eye and head movements, gestures, and a human-like voice. Therefore, researchers have become interested in the question of whether humanoid robots could play an effective role in ASD interventions. On the one hand, humanoid robots could offer many suitable solutions for the improvement of various abilities, including social behaviors (Damm et al., 2013), language (Kim et al., 2013), and imitation (Cook et al., 2014), and for encouragement of the reduction of stereotyped behaviors (Shamsuddin et al., 2013). Researchers have found that “ASD individuals (have), toward robots, behaviors that (typically developing) individuals normally (have) toward human agents” (Pennisi et al., 2016).

On the other hand, robots seem to exert some negative effects on children’s interactional behaviors. Anzalone et al. (2014) built an experimental platform for human-robot interaction using a commercial humanoid robot Nao, and reported school-aged children with ASD exhibited decreased joint attention with Nao, whereas their performance was similar to that of age-matched typically developing (TD) participants when interacting with a human. Bekele et al. (2014) found that participants with ASD needed more prompts in a robot condition compared to a human condition in order to successfully find a target that an agent turned toward. Nevertheless, Warren et al. (2015) demonstrated that the level of prompting required decreased after four robot intervention sessions, which meant that a robot could improve performance in responding to joint attention in people with ASD. Therefore, it remains an open question whether and how humanoid robots could improve the acquisition of joint attention skills in children with ASD.

The key problem here is whether a humanoid robot could function as a better joint attention initiator for the children with ASD; in other words, whether children with ASD can better understand the logic of joint attention as induced by a humanoid robot. The use of eye-tracking could provide an accurate measure of the distribution of attention in a joint attention context (Falck-Ytter et al., 2012; Thorup et al., 2016). Damm et al. (2013) found that children with ASD spent more time on robot face than human face in a joint attention task. However, fixation time could not fully depict if children with ASD could understand robot’s joint attention cues. The fixation time as well as gaze transition analysis were a static description of the amount of attention distribution in and between area of interests (AOIs), which was unable to represent the dynamic attention process.

In recent years, new method was induced to quantify the temporal pattern of the dynamic attention process. Mavadati et al. (2014) utilized a sequence-based Variable-order Markov Model (VMM) to analyze the temporal gaze directional pattern of children with ASD when interacting with a robot in social context, and the results demonstrated that different VMM models were needed to represent the gaze patterns of TD when they were in different conversational roles, similar VMM model, however, could fit the gaze responses of children with ASD when they were in different roles. This research showed that sequence-based method was able to reveal the subtle differences of eye movement patterns between ASD and TD. In the present study, we adopted eye-tracking to investigate whether a humanoid robot (“robot Nao”) could provide better joint attention cues for children with ASD and TD children between 3 and 6 years old. Beyond the analysis of fixation time and gaze transitions, we employed a new algorithm extracting the longest common subsequence (LCS), which allowed for a comparison of the complexity of transition distributions between areas of interest (Krejtz et al., 2015). Using the LCS algorithm, the similarity between the eye movement sequences of participants and a reference sequence, which reflects the underlying logic of joint attention, can be calculated by traversing the two sequences. The algorithm is proposed to quantify how the participant’s gaze dynamically follows the underlying logic of the videos.

Current research taken the advantage of combined fixation and gaze transition analysis, and a sequence-based LCS analysis, and attempted to figure out the eye movement pattern of children with ASD responding to the robot’s joint attention. Based on the findings from previous studies, we hypothesized that (1) children with ASD would spend less time than TD children looking at the (human or robot) agent’s face and at the target; (2) the robot would facilitate joint attention behavior in a similar way to the human; (3) children with ASD would make fewer gaze transitions between the face and the target than would TD children; and (4) children with ASD would show less similarity between eye movement sequence and the reference sequence than the TD group.

## Materials and Methods

### Participants

In total, 21 children with ASD and 22 TD children participated in this study. The children with ASD were recruited from a special education institution for children with ASD. All the children with ASD had a formal diagnosis obtained at a certified hospital by a professional pediatrician or psychiatrist based on the *Diagnostic and Statistical Manual of Mental Disorders, 4th Edition, Text Revision* (American Psychiatric Association, 2000). All TD children were recruited from a local kindergarten and had no history of ASD or other developmental disorders. Both groups were residents of two nearby suburban districts. Three children with ASD and five TD children withdrew from participation in the experiment as a result of reluctance to attend the experiment or for technical reasons. Additionally, data from three children with ASD and two TD children who provided more than two trials in which less than 20% of their total fixation time was on the screen were also excluded from data analysis. Thus, the final participant sample consisted of 15 children with ASD (13 boys and two girls) and 15 TD children (12 boys and three girls). The percentage of total fixation time spent on the screen was similar between the two groups and between the two conditions.

The two groups were matched on chronological age and gender ratio (see Table 1). All the participants had normal vision and hearing, and none had any other conditions or were taking any medications that could influence the results. The receptive language ability of the participants was assessed using the Chinese version of the Peabody Picture Vocabulary Test (PPVT; Biao and Xiaochun, 1990). The TD group had significantly higher PPVT scores than the ASD group, so the PPVT score was treated as a covariate in further analyses. The parents of all participants provided informed and written consent for their participation prior to the experiment. The study protocol was approved by the Ethics Committee of the authors’ affiliated University.

**Table 1:** Demographic details of the final sample.

	ASD	TD		*p*
Age (years)	4.96 ± 1.10	4.53 ± 0.90	*t* = 1.16	0.257
PPVT score	97.65 ± 24.64	119.40 ± 14.42	*t* = 2.95	0.006^∗^
Male:female ratio	13:2	12:3	*χ*^2^ = 0.24	0.624
Total fixation time (%)	43.57 ± 20.22	47.38 ± 14.22	*t* = -0.60	0.556

*ASD, autism spectrum disorders; TD, typically developing; PPVT, Peabody Picture Vocabulary Test. ^∗^p < 0.05.*

### Stimuli

The stimuli fell into two conditions: (1) a human condition, in which a man was displayed sitting behind a table with a neutral facial expression and attempted to induce joint attention by turning his head toward one of three toy trucks positioned in front of him; and (2) a robot condition, in which a social robot was displayed in the same setting and carrying out the same actions. The three toys were located on the left side, in the middle, and on the right side of the table. In addition, there was a picture frame in the background, which played the role of a distractor.

**FIGURE 1:**
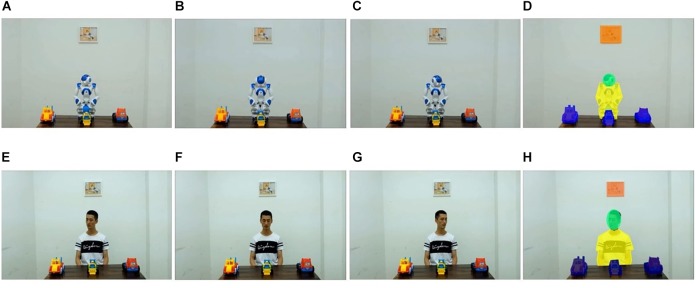
Stills from the stimuli in **(A–C)** the robot condition, and **(E–G)** the human condition. **(D,H)** Indicate the areas of interest: the green area is the agent’s face, the yellow area is the agent’s body, the blue areas are the three objects (target and non-targets), the orange area is the picture frame (distractor), and the rest of the screen is the background. Written informed consent was obtained from the individual for the publication of this image.

Each condition included four video clips, each of which consisted of two segments, a greeting segment and a joint attention segment. In the greeting segment, the agent raised his or its head, looked directly at the participant, and said “Hello, kid” in Chinese (2.5 s). In the joint attention segment, the agent started to turn his or its head toward one of the three toy trucks (i.e., the target object) and kept looking at the target (5–6 s). To enhance the humanoid feature of the robot, the sparks (LED inside) of the robot’s eyes changed twice rapidly before the joint attention segment simulating the human blinks. In all videos, the agent looked at each target in a fixed sequence: left-middle-right-left. Stills from a video clip in each condition are shown in Figure 1.

The robot used in this project was Nao, which is a programmable humanoid robot developed by Aldebaran Robotics Company. The robot is 58 cm tall and has 25 degrees of movement freedom. After careful programming, it was able to carry out the actions described above.

### Procedure

Each participant sat in a child-sized chair in front of a monitor in a quiet classroom, which all participants were familiar with. Before the experiment, the participant had chances to free explore, play and interact with the robot NAO for 10 min, during which the robot would introduce itself, talk to the participants, ask or answer questions, and dance with music. If a participant with ASD was unable to settle down, their parent was instructed to sit behind them. The experimenter first showed a cartoon movie to attract the participant’s attention; after the participant had watched these cartoon clips quietly for more than 1 min, a 5-point eye-tracking calibration procedure was carried out. For accurate calibration, participants were required to fixate within 1° of each fixation point during this procedure. Subsequently, the formal experiment started. The videos were displayed on a 22″ color LCD monitor at a distance of approximately 60 cm and subtended a visual angle of approximately 32° horizontally and 24° vertically. The screen resolution was 1920 × 1080 pixels. For half of the participants, the human condition was presented first, and for the other half, the robot condition was presented first. The participant was always allowed to move their head position freely during the experiment, but was asked to “sit quite still.” If the participant could not complete the calibration procedure or the experimental session, the same experimental procedure was repeated in the same setting at least 2 weeks later.

Participants’ eye movements were recorded using a remote screen-based Tobii X3-120 eye-tracker system (Tobii, Sweden). The frequency of recording was 120 Hz and locations were accurate to within 1° of the visual angle. This level of accuracy was maintained throughout the experiment as long as the participant kept their eyes within a virtual space measuring 20 × 20 × 20 cm. Moving outside the virtual space could cause recording to stop temporarily, and recording could be restarted by returning the head to the correct position.

### Data Analysis

#### Definition of AOIs

Only data collected during the joint attention stage were entered into the analyses. Using the AOI tool in the Tobii Studio software, we defined 7 AOIs, namely the agent’s face, the agent’s body, each of the three toys, the picture frame, and the background (see Figure 1). Gaze data was extracted for each AOI.

#### Fixation Analysis

A fixation was defined as an event in which eye-tracking data indicated that the participant’s gaze remained within 1.5° of the visual angle of a given location for 100 ms or longer. The first fixation time for a given AOI was defined as the latency of the first fixation within this AOI, and the percentage fixation duration was defined as the proportion of the total time of fixation on the screen spent within a given AOI. The toy that the agent looked at was defined as the target, and the other two toys were defined as non-targets. The average percentage fixation durations of the target and the non-targets across the whole experiment were computed, the 2 non-target toys were then averaged into one AOI. So 6 AOIs were included in the analysis: the agent’s face, the agent’s body, the target, non-targets, the picture frame, and the background.

#### Gaze Transitions

**FIGURE 2:**
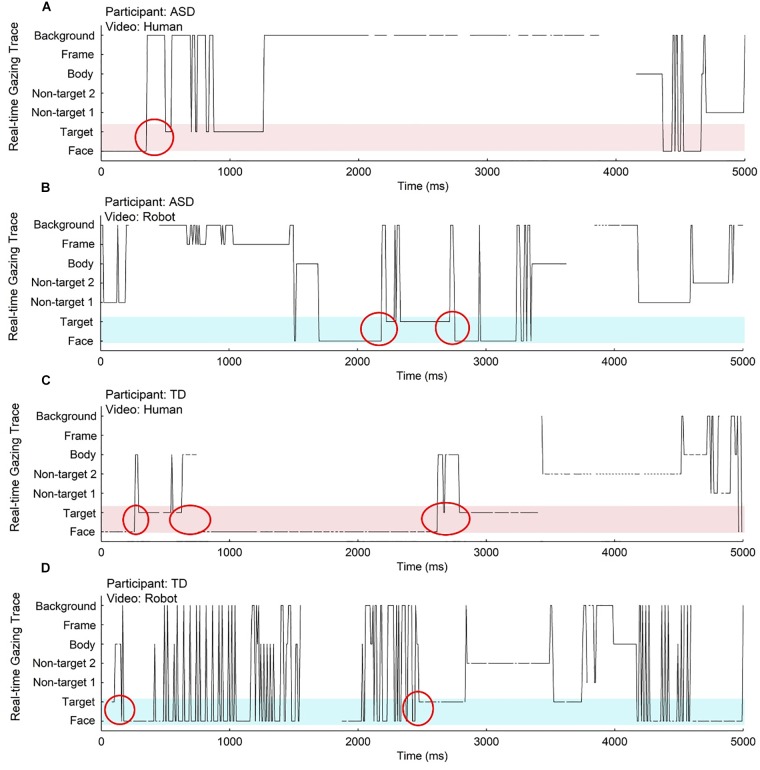
The real-time gaze traces of two participants, one child with ASD **(A,B)** and one TD child **(C,D)** during the joint attention segment of videos in each condition (human condition: **A,C**; robot condition: **B,D**). Different areas of interest are represented on the *y* axis, and the *x* axis represents time. Black lines represent eye movement traces, where each point corresponds to a fixation in one area of interest recorded at a certain time point. Red circles indicate gaze transitions between the agent’s face and the target. Light red and light blue shaded areas cover the face and target regions, which are used in the reference sequences in LCS analysis; joint attention behavior can be observed in these areas.

The number of gaze transitions between the agent’s face and the three toys was extracted using a tailor made script written in Matlab and Java. Transitions between the face and the target were defined as congruent gaze shifts (Figure 2), and transitions between the face and a non-target were defined as incongruent gaze shifts. The analysis focused directly on the number of congruent and incongruent gaze shifts.

#### LCS Analysis

The LCS algorithm has been widely used in computational biology and human genome sequencing. In general, for two sequences *X* = (*X*_1_, *X*_2_,…,*X*_n_) and *L* = (*L*_1_, *L*_2_,…,*L*_i_), if *L* = (*X*_j_1__, *X*_j_2__,…*X*_j_i__, …), where 1 ≤ *J*_1_<*J*_2_<…<*J*_i_<… ≤ *J*_n_, then *L* is a subsequence of *X*. For any two sequences *X* and *Y*, if *L* is a subsequence of *X* and *Y*, and there is no other common subsequence of *X* and *Y* longer than *L*, then *L* is the LCS of *X* and *Y*. The LCS of two given strings is not necessarily unique. Nevertheless, the length of the LCS is unique.

During the joint attention stage of the eye-tracking recordings, it was assumed that the participant should focus on either the agent’s face or the target at any given moment. The reference sequence defined in the present study reflected the intrinsic logic of the stimuli, namely that the participant should initially focus on the agent’s face, follow the direction of their gaze, and then look at the target, possibly moving back and forth between these regions. Thus, we formulated a reference sequence *Y* corresponding to this pattern. Each element in the sequence *Y* was a set containing two values, representing the agent’s face and the target, respectively. Besides, *X* is the sequence abstracted from the participant’s eye-tracking recordings. Each element in the sequence *X* represented the value corresponding to the AOI that the participant gazed. *N* was defined as the length of *Y*, which was the same as the length of *X*; thus, both *X*_i_ and *Y*_i_ corresponded to gaze locations at the same time point. The LCS algorithm applied in this case was based on a recursive function. A two-dimensional matrix *M*[*i,j*] was derived according to Equation (1) with initial values set as 0. The length of the LCS of *X* and *Y* was therefore equal to the element of *M* with the largest value. The proportions presented in the Results section on LCS scores are the ratio of the length of the LCS of *X* and *Y* to the total length of *X* (or *Y*).

(1) $$M[i,j]=\left\{ \begin{array}{c} 0,if\;i=0\;or\;j=0\\ M[i-1,j-1]+1,\;if\;i,j>0\;and{\text{ X}}_{\text{i}}\subseteq {\text{Y}}_{\text{j}}\\ Max\{M[i-1,j],M[i,j-1]\},\;if\;i,j>0\;and{\text{ X}}_{\text{i}}\subseteq {\text{Y}}_{\text{j}} \end{array}\right.$$

The measure LCS score can record any number of the back and forth movements and the flexible fixation time of the agent’s face or the target during the movements. It could also be taken into account even when the gazing to the agent’s face or the target is not continuous, i.e., the participant looked at the other AOIs during the back and forth movements. On the other hand, it should be noted that if the participant performs good joint attention it is necessary to have a high score of the LCS analysis. However, a high LCS score is not sufficient to show good joint attention performance. Therefore, it would be good that the LCS analysis was performed together with the other analyses to verify the joint attention tasks.

#### Access to Materials

All the research materials described above may be obtained by contacting the corresponding authors.

## Results

For all the dependent variables mentioned above, a repeated measure analysis of variance (ANOVA) was conducted to determine the effects of the independent variables. Group (ASD vs. TD) was a between-participants variable; stimulus type (human vs. robot condition) and AOI (face, body, target, non-target, picture frame, or background) were within-participants variables. *Post hoc* analysis was conducted if a main effect or an interaction was significant. Statistical analyses were performed using SPSS 20.0.

### First Fixation Time

**FIGURE 3:**
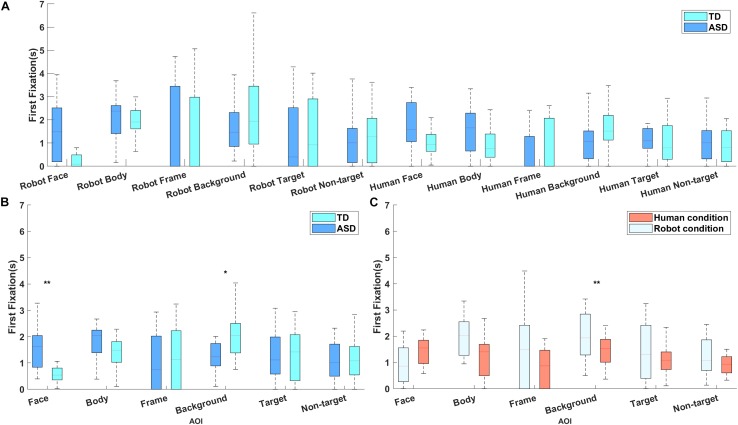
**(A)** The latency (s) of participants’ first fixation on different areas of interest. **(B)** Results of *post hoc* analysis of the interaction between group and area of interest, indicating that the typically developing group looked at the face earlier than did the group with ASD, while the group with ASD looked at the background earlier than did the typically developing group. **(C)** The interaction between stimulus type and area of interest, demonstrating that all participants looked at the human body earlier than they looked at the robot body. ^∗∗^Means that the significant level is *p* < 0.01 and ^∗^ means *p* < 0.05.

To determine whether both groups were equally likely to be looking at the agent’s face before an initial gaze shift occurred, a repeated measures ANOVA was conducted over the first fixation time for each AOI. There were significant main effects of stimulus type [*F*(1, 28) = 5.502, *p* = 0.026, partial *η*^2^ = 0.164] and of AOI [*F*(1, 28) = 2.989, *p* = 0.014, partial *η*^2^ = 0.096], and significant interactions between group and AOI [*F*(5, 28) = 3.164, *p* = 0.010, partial *η*^2^ = 0.102] and between stimulus type and AOI [*F*(1, 28) = 2.769, *p* = 0.020, partial *η*^2^ = 0.090; Figure 3A]. *Post hoc* analysis revealed that participants looked earlier at the human agent than at the robot (*p* = 0.026), and that the TD group looked at the agent’s face earlier than did the ASD group (*p* = 0.001), but spent more time fixating on the background (*p* = 0.049; Figure 3B). Moreover, participants looked earlier at the human body than at the robot body (*p* = 0.008; Figure 3C). All these effects remained significant when PPVT score was included as a covariate. No other main effects or interactions reached significance.

### Proportion of Total Fixation Time

**FIGURE 4:**
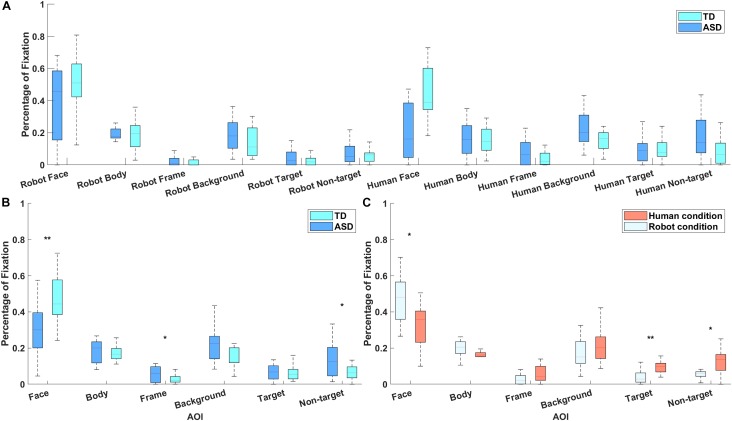
**(A)** The percentage of fixation time spent on different areas of interest by participants with ASD and TD participants. **(B)** Results of *post hoc* analysis of the interaction between group and area of interest, indicating that the TD group fixated longer than the group with ASD on the agent’s face, whereas the ASD group looked for longer than the TD group at the picture frame and non-targets. **(C)** The interaction between stimulus type and area of interest, demonstrating that all participants spend more time looking at the face area in the robot condition (*p* = 0.038), and more time looking at the target and non-targets in the human condition. ^∗∗^Means that the significant level is *p* < 0.01 and ^∗^ means *p* < 0.05.

A repeated measures ANOVA revealed a significant main effect of AOI [*F*(5, 28) = 44.00, *p* < 10^-7^, partial *η*^2^ = 1.000], and significant interactions between AOI and group [*F*(5, 28) = 5.63, *p* = 10^-3^, partial *η*^2^ = 0.946] and between AOI and stimulus type [*F*(5, 28) = 3.08, *p* = 0.03, partial *η*^2^ = 0.684; Figure 4A]. The main effects of group and stimulus type, the interaction between group and video type, and the three-way interaction did not reach statistical significance. *P*-values and power levels were adjusted using the Huynh-Feldt epsilon method. *Post hoc* analysis (with Bonferroni correction) showed that the TD group fixated longer than the ASD group on the agent’s face (*p* = 0.002), whereas the ASD group looked longer than the TD group at the picture frame (*p* = 0.045) and the non-targets (*p* = 0.036; Figure 4B). Both participant groups spent more time looking at the face area in the robot condition (*p* = 0.038), and more time looking at the target (*p* = 0.004) and non-target (*p* = 0.045) in the human condition (Figure 4C). However, when PPVT score was included as a covariate, only the interaction between AOI and group remained significant [*F*(5, 28) = 5.02, *p* = 0.02, partial *η*^2^ = 0.157].

### Gaze Transition

**FIGURE 5:**
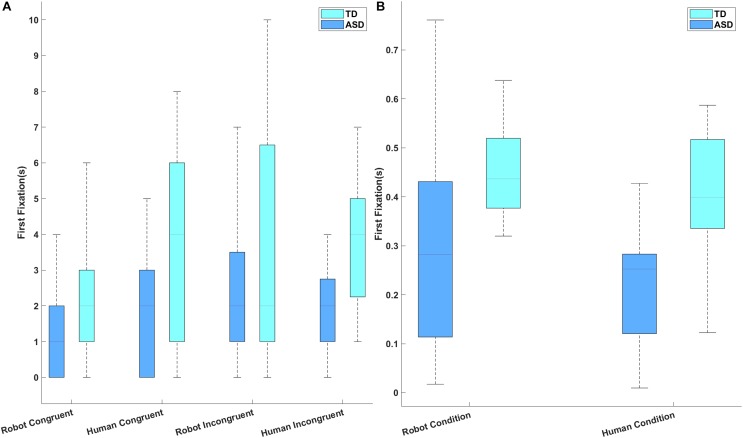
**(A)** The number of congruent and incongruent gaze transitions made by each participant group for each stimulus types. **(B)** Longest common subsequence (LCS) scores for each participant group and stimulus type.

A 3 × 2 ANOVA (group, stimulus type, target) did identify a significant difference between the two groups on the number of gaze transitions [*F*(1, 28) = 9.282, *p* = 0.005, partial *η*^2^ = 0.154], but there was no significant main effect of stimulus type [*F*(1, 28) = 1.098, *p* = 0.304, partial *η*^2^ = 0.038] or target [*F*(1, 28) = 1.844, *p* = 0.185, partial *η*^2^ = 0.062; Figure 5A]. Furthermore, there was no interaction between group and stimulus type [*F*(1, 28) = 0.962, *p* = 0.335, partial *η*^2^ = 0.033], between group and target [*F*(1, 28) = 0.014, *p* = 0.908, partial *η*^2^ < 0.001], between stimulus type and target [*F*(1, 28) = 1.196, *p* = 0.283, partial *η*^2^ = 0.041], or between group, stimulus type, and target [*F*(1, 28) = 0.159, *p* = 0.699, partial *η*^2^ = 0.007]. The TD children made a significantly higher number of congruent gaze transitions than did the children with ASD; this main effect survived after controlling for the influence of PPVT score [*F*(1, 28) = 8.335, *p* = 0.008, partial *η*^2^ = 0.236].

### LCS

A repeated measures ANOVA revealed a significant main effect of group [*F*(1, 28) = 11.18, *p* = 0.002, partial *η*^2^ = 0.898; Figure 5B]. *Post hoc* analysis revealed that the TD children obtained significantly higher LCS scores than did the children with ASD, which implies that TD children were better able to follow the logic of the videos. This effect remained significant when PPVT score was included as a covariate [*F*(1, 28) = 8.710, *p* = 0.006, partial *η*^2^ = 0.244]. The main effect of stimulus type [*F*(1, 28) = 1.852, *p* = 0.184, partial *η*^2^ = 0.062] and the interactions between stimulus type and group [*F*(1, 28) = 0.913, *p* = 0.347, partial *η*^2^ = 0.032] were not statistically significant.

## Discussion

Although robot-based intervention is a promising tool for people with ASD, little was previously known about how children with ASD respond to the direction of a robot’s gaze and head direction. In the present study, a commercial social robot was used along with eye-tracking technology to obtain evidence on the effect of using a robot on the joint attention behavior of children with ASD.

### Joint Attention Deficits in Children With ASD

First, the present research confirmed that children with ASD did show deficits in joint attention. Participants with ASD made fewer gaze transitions between the joint attention initiator and the target than did the TD group. The reason for this outcome may be that children with ASD have difficulty capturing and utilizing the social meaning of faces, leading to an atypical distribution of attention toward the face in both conditions. This interpretation is supported by the fact that the group with ASD fixated later and for less total time on the agent’s face, and earlier and for longer on the picture frame and non-target objects, compared to the TD group. Previous studies have indicated that people with ASD exhibit atypical face-scanning patterns, particularly making fewer fixations in the eye regions, and this visual pattern affects the extraction of social information from faces (Chawarska and Shic, 2009). The LCS findings in the present study also demonstrate that participants with ASD were less able than TD participants to follow the underlying logic of the stimuli. Thus, the current study demonstrated this difficulty experienced by the ASD participants at a behavioral level.

### The Effect of a Robot Initiator on Joint Attention

The present study indicated that participants showed more interest in the robot’s face than the human’s face, but spent more time looking at the target and non-targets in the human condition. These findings could be interpreted in two ways. First, this pattern of results could indicate that the robot was able to successfully attract the attention of the participants. The results described above indicated that participants with ASD tended to avoid or ignore the human’s face, which could somewhat account for the atypical social attention observed in people with ASD. It has always been a difficult task to ensure that children with ASD focus on faces and the social signals associated with them during diagnosis and intervention. Making use of a robot, however, could benefit from the unique advantages of such an agent and may catch more focuses of the children with ASD during the diagnosis and interventions. Damm et al. (2013) found that people with ASD engage in more eye contact with and spend longer fixating on a robot face than a human face. A neuroimaging study has also reported similar brain activation patterns in participants with ASD and TD participants when processing a robot face (Jung et al., 2016). Second, the robot seemed to affect the distribution of attention toward the target. Previous research has found that people with ASD focus their attention on the robot instead of the target object in this type of task (Robins and Dautenhahn, 2006), which seems to imply that the robot might act as a distractor in the joint attention process. Nevertheless, our results also indicated that participants performed similarly in gaze transition and LCS scores in the robot condition and the human condition. It is plausible to assume that the robot might exert a negative effect on the amount of time spent fixating on the target, but the robot could still prompt gaze transitions in the same way as the human agent, and participants with ASD could be equally able to understand the social logic of the situation in the robot condition.

Although current research does not address the potential clinical application of robots in an ASD intervention directly, the findings may demonstrate that, compared to human, the present robot NAO is not an ideal joint attention agent in intervention for children with ASD, but this does not deny the role of robot. The present study indicates that people with ASD can understand the logic induced by the robot. Furthermore, a previous research has also indicated that similar cerebral areas were activated in people with ASD when interacting with a robot compared to those activated in TD controls when interacting with a human agent (Chaminade et al., 2012). These outcomes imply that a robot might be suitable social partner and mediator for children with ASD. Using a carefully designed environmental setting and procedure, interactions between the robot and a child with ASD could help the child to understand the social logic underlying interaction, and this understanding could be extended to real life contexts (Pennisi et al., 2016). Certainly, however, there is no suggestion that interaction with a robot could function as a replacement of interaction with a human. Although questions remain regarding the limitations of potential robot applications in longitudinal interventions, the use of robots has the potential to emerge into the present system of ASD interventions, and for robots to take on roles as social mediators between people with ASD and therapists, which would provide great relief in those countries with a shortage of professionals (Coeckelbergh et al., 2016).

### The LCS Analysis

The result of fixation analysis and the LCS analysis in the present study differed slightly; two potential explanations may account for this. Essentially, a single fixation represents the sum of the participant’s gaze location over several continuous time points, and the fixation time on a particular AOI was the sum of all fixations within that AOI. In contrast, the LCS measure involved direct analysis of the similarity between two eye movement sequences, which contained spatial and temporal information about eye movement, and may therefore be assumed to better describe both the static fixation and dynamic gaze transition of eye movements. In the present study, the predefined reference sequence was relatively simple, leading to LCS scores that were equivalent to the sum of the time spent on looking at the agent’s face and the targets. However, the LCS method itself is universal and could be applied to much more complex situations. The reference sequence could be modified accordingly for joint attention stimuli using different underlying logic, and the method could be applied to real social scenes involving more complicated logic. Shic et al. (2008) used a similar entropy-based methodology to measure the eye movements of participants with ASD in response to static pictures, and obtained results that went beyond the traditional fixation analysis, which indicates that this approach could be a promising tool for future use.

### Limitations

This study had several limitations. First, despite its humanoid appearance, the robot Nao was incapable of fully conveying the same social and emotional cues as a human, for example the eyes of robot could not generate gaze shift cues without the head turning. On the other hand, the eyes of robot Nao could sparkle with colorful LEDs, which provide highlight of eyes area that draw attention of children. To eliminate the influence of such factors, our experimental design aimed to minimize the use of language, and the actor filmed for the videos in the human condition was asked to keep his expression neutral throughout the whole process. This could affect the ecological validity of the stimuli, and should be mitigated by implementing in-person interactions rather than video stimuli in the future. Second, the LCS algorithm has a potential to produce better characterizations of joint attention behavior. The current approach did not take gaze latency into consideration, and moreover, this version of the algorithm still could not capture differences in the details between conditions. Doing so would require different weightings for different eye movement patterns. Finally, the present research focused only on the performance of children with ASD in a single experimental session. Thus, the findings offer limited information on the potential responses of children with ASD to a complete intervention course. This limitation is shared by many other studies. Further clarification is needed on how a robot-based intervention might be integrated with generally accepted principles to formulate an intergrative and systematic intervention process.

## Data Availability

All datasets generated for this study are included in the manuscript and/or the supplementary files.

## Ethics Statement

The study protocol was approved by the Ethic Committee of South China Normal University.

## Author Contributions

WC collected and analyzed the data, and wrote the manuscript. WS analyzed the data and wrote the manuscript. XL collected the data and provided the materials. SZ analyzed the data. GZ and YW collected the data. SH provided the financial support. HZ and JC designed the experiment and data analysis method, and reviewed the manuscript.

## Conflict of Interest Statement

The authors declare that the research was conducted in the absence of any commercial or financial relationships that could be construed as a potential conflict of interest.
